# Genotype–phenotype spectrum and clinical outcomes of glycogen storage disease type I: A 15-year experience at Vietnam National Children's Hospital

**DOI:** 10.1016/j.ymgmr.2026.101337

**Published:** 2026-07-10

**Authors:** Hang Thi Nguyen, Dung Chi Vu, Tung Viet Cao, Van Khanh Tran, Thao Phuong Bui, Ngoc Bich Thi Can, Lan Hoang Nguyen, Ngoc Lan Nguyen, Thinh Huy Tran, Nguyen Huu Tu, Le Thi Phuong, Khanh Ngoc Nguyen

**Affiliations:** a Hanoi Medical University, Hanoi, Viet Nam; b Center of Endocrinology, Metabolism, Genetic/Genomics and Molecular Therapy, Vietnam National Children's Hospital, Hanoi, Viet Nam; c Department of Pediatrics, University of Medicine and Pharmacy, Vietnam National University, Hanoi, Viet Nam; d Vietnam National Children's Hospital, Hanoi, Viet Nam; e Vietnam University of Traditional Medicine, Hanoi, Viet Nam; f Center for Gene and Protein Research, Hanoi Medical University, Hanoi, Viet Nam; g Biochemistry Department, Hanoi Medical University, Hanoi, Viet Nam; h Thai Nguyen University of Medicine and Pharmacy, Thai Nguyen, Viet Nam

**Keywords:** Glycogen storage disease type I, *G6PC* variants, *SLC37A4* variants

## Abstract

**Background:**

Glycogen Storage Disease Type I (GSD I) is an inherited metabolic disorder characterized by impaired hepatic glucose production due to defects in gluconeogenesis and glycogenolysis. Two subtypes are recognized: GSD Ia (*G6PC*) and GSD Ib (*SLC37A4*). Genotype–phenotype correlations and long-term outcomes in Southeast Asia remain insufficiently characterized. This study aimed to describe the clinical, biochemical, and molecular features of Vietnamese children with GSD I and evaluate treatment outcomes***.***

**Methods:**

Twenty-four patients from 19 families diagnosed at the Vietnam National Children's Hospital between 2016 and 2025 were included. All underwent genetic testing. Clinical characteristics and laboratory parameters were collected at diagnosis and during follow-up***.***

**Results:**

Twelve distinct variants were identified, including eight in *G6PC* and four in *SLC37A4*, with one novel variant (c.1193G > A). The most common *G6PC* variants were c.518 T > C, c.356 A > T, and c.648G > T, while c.706_708delGTG predominated in *SLC37A4*. The median age at symptom onset was 4 months, and 70% of patients presented with hepatomegaly. Elevated liver enzymes and lactate levels were observed in all cases, and 88.2% had hypertriglyceridemia. Dietary adherence was generally poor despite uncooked cornstarch therapy. During follow-up, patients with GSD Ia developed complications including short stature, hepatic adenoma, pancreatitis, hypertension, kidney stones, and cirrhosis, while all GSD Ib patients experienced neutropenia and required empagliflozin for recurrent infections***.***

**Conclusion:**

Hepatomegaly with elevated liver enzymes, lactate, and triglycerides is a common presentation of GSD I. Variant distribution may differ across populations, and complications can occur at any age.

## 1 Introduction

Glycogen Storage Disease Type I (GSD I), also referred to as von Gierke disease, is a rare autosomal recessive metabolic disorder caused by defects in the final steps of glucose production from glycogenolysis and gluconeogenesis. These defects impair the hydrolysis or transport of glucose-6-phosphate, thereby preventing the normal release of free glucose into the bloodstream [1], [2]. The estimated global incidence of GSD I is approximately 1 in 100,000 live births [3]. At the molecular level, GSD I is classified into two major subtypes. GSD Ia (OMIM #232200) arises from pathogenic variants in the *G6PC* gene, which encodes the enzyme glucose-6-phosphatase. In contrast, GSD Ib (OMIM #232220) results from pathogenic variants in the *SLC37A4* gene, encoding the glucose-6-phosphate transporter [2], [4]. Deficiencies in these proteins disrupt endogenous glucose production, particularly during fasting, leading to a metabolic profile characterized by severe hypoglycemia, hepatomegaly, lactic acidosis, hyperuricemia, and hyperlipidemia [5], [6]. Beyond the metabolic manifestations shared with GSD Ia, patients with GSD Ib frequently develop immune-related complications. Defective neutrophil function leads to chronic neutropenia, which predisposes patients to recurrent infections, oral ulcers, and inflammatory bowel disease–like colitis. These complications further complicate dietary management and contribute substantially to the long-term disease burden [7], [8].

Advances in molecular diagnostic technologies, particularly the widespread adoption of Next-Generation Sequencing (NGS), have greatly improved the accuracy of GSD I diagnosis and enabled precise subclassification into GSD Ia and GSD Ib. To date, more than 170 pathogenic or likely pathogenic variants have been reported in *G6PC*, including missense, nonsense, frameshift, and splice-site mutations (https://www.hgmd.cf.ac.uk/ac/gene.php?gene=G6PC). Population-specific variant distributions have also been described. For example, c.247C > T (p.Arg83Cys) and c.1039C > T (p.Gln347Ter) are frequently observed among White populations, whereas c.379_380dupTA (p.Tyr128ThrfsTer3) and c.247C > T (p.Arg83Cys) are common in Hispanic cohorts [9], [10]. In East Asian populations, c.648G > T (p.Leu216Leu) is among the most prevalent variants, with c.648G > T accounting for approximately 91% of mutant alleles in Japan and around 75% in Korea [9], [10], [11], [12]. Among Ashkenazi Jewish populations, c.247C > T (p.Arg83Cys) represents nearly 98% of reported variants [13]. Similarly, more than 150 pathogenic/likely pathogenic variants have been identified in the *SLC37A4* gene as causes of GSD Ib, encompassing missense, nonsense, frameshift, and splice-site variants (https://www.hgmd.cf.ac.uk/ac/gene.php?gene=SLC37A4). In White populations, c.1015G > T (p.Gly339Cys) and c.1042_1043delCT (p.Leu348ValfsTer53) are among the most frequently reported variants, while c.352 T > C (p.Trp118Arg) accounts for nearly 50% of mutant alleles in Japanese patients [10], [14], [15].

Management of GSD I primarily relies on nutritional therapy aimed at maintaining stable blood glucose levels and preventing metabolic decompensation. Patients are typically advised to consume frequent meals and avoid prolonged fasting. Slow-release carbohydrate sources, particularly uncooked cornstarch (UCCS) or extended-release cornstarch formulations such as Glycosade, are widely used to sustain normoglycemia. Dietary intake of fructose, sucrose, and lactose is also restricted [16]. In infants and young children whose glucose levels remain unstable despite UCCS therapy, continuous enteral feeding may be required as an additional nutritional strategy. In such cases, nocturnal glucose delivery through a nasogastric tube or gastrostomy provides a continuous carbohydrate supply during sleep and helps prevent fasting hypoglycemia [16].

The effectiveness of treatment is monitored through periodic clinical evaluation, laboratory testing, and imaging studies. The primary goals of long-term management are the prevention of hypoglycemia, support of normal growth, and reduction of complications such as hepatic adenoma, hepatocellular carcinoma, hypertension, renal disease, pancreatitis, developmental delay, neutropenia, and recurrent infections [16], [17].

Although molecular diagnosis allows accurate disease classification, genotype–phenotype correlations in GSD I remain incompletely understood, particularly regarding disease severity, complication risk, and therapeutic response. These knowledge gaps are especially pronounced in Southeast Asia, where available evidence is largely limited to small case series [18], [19]. Given the scarcity of integrated clinical and genetic datasets from this region, further studies are required to better characterize the molecular spectrum and clinical outcomes of affected patients. The present study, therefore, investigates the genotype–phenotype spectrum and treatment outcomes of children with GSD I treated at Vietnam National Children's Hospital, with the aim of improving understanding of disease characteristics and management in an underrepresented Southeast Asian population.

## 2 Materials and methods

### 2.1 Study design and participants

This study was designed as a single-center retrospective cohort investigation conducted at the Center for Endocrinology, Metabolism, Genetics, and Molecular Therapy, Vietnam National Children's Hospital (VNCH). Medical records were reviewed for pediatric patients younger than 18 years who had received a molecular diagnosis of glycogen storage disease type I (GSD I) between January 2016 and November 2025. Patients were excluded if they had other hepatic glycogen storage disorders, including GSD III, VI, or IX, or if their medical records lacked sufficient clinical or laboratory information for analysis.

### 2.2 Genetic analysis

Genomic DNA was extracted from whole-blood samples using the QIAamp DNA Blood Kit (Qiagen, Hilden, Germany). Genetic testing was conducted using a comprehensive glycogen storage disease panel that was described in the previous [20]. Variant classification and interpretation were performed using publicly available population and disease databases (e.g., gnomAD, dbSNP, OMIM, ClinVar), the American College of Medical Genetics and Genomics (ACMG) classification system [21] in conjunction with internal laboratory data. When parental samples were available, segregation analysis was performed by Sanger sequencing to verify inheritance patterns of the identified variants.

### 2.3 Clinical and laboratory evaluation

Clinical and biochemical information was collected at the initial clinical evaluation, including anthropometry, medical history, physical examination, operational definitions, laboratory tests, and image findings. The complications were documented throughout follow-up.

#### 2.3.1 Anthropometric assessment

Anthropometric measurements included body weight, height or length, body mass index (BMI), and head circumference. Measurements were obtained using calibrated Seca equipment (Seca GmbH & Co. KG, Hamburg, Germany). Children aged ≤2 years were measured in the recumbent position, whereas those older than 2 years were measured standing. *Z*-scores were calculated according to the 2006/2007 World Health Organization growth standards.

#### 2.3.2 Medical history

Relevant clinical history included birth weight, neonatal hypoglycemia, seizure episodes, developmental milestones, and family history of metabolic disease or unexplained sibling deaths.

#### 2.3.3 Physical examination

Clinical examination focused on identifying hepatomegaly, splenomegaly, abdominal collateral circulation, xanthomas, anemia, and manifestations suggestive of hyperuricemia. Hepatomegaly was defined as a palpable liver edge extending more than 2 cm below the costal margin on physical examination in children, consistent with guidance from the American Academy of Pediatrics [22]. Systemic hypertension was defined according to age-specific pediatric blood pressure criteria from the 2017 American Academy of Pediatrics guideline [23], based on repeated blood pressure measurements exceeding the 95th percentile for age, sex, and height, or ≥ 130/80 mmHg in patients aged ≥13 years.

### 2.4 Operational definitions

Hypoglycemia was defined as a plasma glucose concentration < 2.8 mmol/L, while neutropenia was defined as an absolute neutrophil count (ANC) <1.5 × 10^9^/L. Serum lipid parameters included total cholesterol (TC), low-density lipoprotein cholesterol (LDL-C), high-density lipoprotein cholesterol (HDL-C), and triglycerides (TG). Reference ranges were as follows: TC: 2.88–5.23 mmol/L; LDL-C: <3.4 mmol/L; HDL-C: 0.9–1.79 mmol/L; TG: 0.51–2.38 mmol/L. Serum uric acid concentrations were measured in μmol/L, with age- and sex-specific reference ranges defined as: 0–<15 days: 170–739; ays–<1 year: 103–374; 1–<12 years: 114–290; 12–<19 years: 160–347 (female) and 162–448 (male). Hyperlactatemia was defined as a serum lactate level > 2 mmol/L. Normal reference values for liver enzymes were 20–40 U/L for aspartate aminotransferase (AST/GOT) and 5–40 U/L for alanine aminotransferase (ALT/GPT). The normal arterial blood pH range was 7.35–7.45.

#### 2.4.1 Laboratory investigations

Laboratory assessments included complete blood count (CBC), absolute neutrophil count (ANC), glucose, lactate, triglycerides, cholesterol, uric acid, urea, creatinine, AST, and ALT. Blood samples were obtained during fasting conditions. Biochemical parameters were measured photometrically using an AU 5800 automatic biochemical analyzer.

#### 2.4.2 Imaging studies

Imaging evaluations included abdominal ultrasonography, computed tomography, echocardiography, and electrocardiography (ECG).

### 2.5 Treatment and follow-up management

Treatment strategies followed contemporary international recommendations available during the study period, including European guidelines for glycogen storage disease type I [24], and ACMG nutrition management guidelines [16]. Dietary management aimed to prevent fasting hypoglycemia and maintain metabolic stability through frequent feeding, restriction of fructose-, sucrose-, and galactose-containing foods, and supplementation with uncooked cornstarch (UCCS). UCCS therapy was generally initiated after 12 months of age, and the UCCS dosage used was 1–1.5 g/kg every 3–4 h for young children and 1.5–2 g/kg every 4–5 h for older children and adolescents. UCCS is mixed with cold water or unsweetened beverages. The diet was designed to ensure adequate energy intake according to age, with a macronutrient distribution of 60% carbohydrates, 15% protein, and 25% lipids. Carbohydrates included sources from UCCS. The amount of carbohydrates in each meal was individualized according to age, weight, physical activity, and metabolic control goals. Patients were advised to limit added sugars and foods high in fructose, sucrose, and galactose, while still allowing fruits and vegetables within the framework of controlling total carbohydrate intake. Unsaturated fatty acids such as olive oil were preferred, and we use medium-chain triglycerides (MCTs) for patients. In addition, patients were supplemented with micronutrients, vitamins, and minerals according to age-related needs to ensure normal development.

Physicians and clinical dietitians jointly developed dietary plans and feeding schedules, adapting recommendations to patients' daily routines, eating habits, school attendance, and family circumstances. During much of the study period, nutritional counseling was primarily provided by general clinical dietitians because specialized metabolic dietitians were unavailable at our center. Continuous nocturnal enteral feeding via nasogastric tube or gastrostomy was not routinely implemented.

Patients were generally scheduled for specialized outpatient follow-up every 3 months. Follow-up assessments included clinical evaluation, biochemical monitoring, growth assessment, and hepatic surveillance using abdominal ultrasonography. Additional investigations, including computed tomography (CT), magnetic resonance imaging (MRI), transient elastography (FibroScan), and other examinations, were performed when clinically indicated. Caregivers were instructed to perform capillary blood glucose monitoring before main meals, at bedtime, and overnight to evaluate fasting tolerance and nocturnal hypoglycemia.

Continuous glucose monitoring (CGM) devices are available in Vietnam; however, national health insurance does not currently reimburse their use for patients with GSD. Glycosade was unavailable in Vietnam throughout the study period. Caregivers received dietary counseling and emergency management education during outpatient visits and hospital admissions, and remote support through telephone consultation or messaging applications was provided when necessary.

Patients with GSD Ib underwent additional monitoring for neutropenia, recurrent infections, oral ulceration, and inflammatory bowel disease-like manifestations. Beginning in September 2024, empagliflozin therapy was introduced for selected patients according to clinical severity and hematologic manifestations. Treatment initiation, dose adjustment, and therapeutic response were retrospectively reviewed from medical records.

Caregivers received dietary counseling and emergency management instructions during outpatient visits and hospital admissions. Remote consultation via telephone or messaging applications was also provided when necessary for dietary adjustment and management during acute illness.

### 2.6 Data analysis

All statistical analyses were performed using SPSS 25.0 (IBM Corp., Armonk, NY). Continuous variables with a normal distribution are presented as mean ± standard deviation (SD), whereas variables with non-normal distributions are expressed as median with interquartile range (IQR). Categorical variables are summarized as frequencies and percentages. Group comparisons for categorical variables were conducted using the chi-square test or Fisher's exact test, as appropriate. Continuous variables were compared using the Mann–Whitney *U* test when distributional assumptions were not met. A *p*-value <0.05 was considered statistically significant.

## 3 Results

### 3.1 Clinical and biochemical characteristics

A total of 24 patients from 19 families were diagnosed with Glycogen Storage Disease Type I and received clinical management at our center. Detailed clinical and biochemical characteristics of the study cohort are summarized in Table 1 and Supplementary Table S1. Within the GSD Ia subgroup, 15 patients from 12 unrelated families were identified. Among them, six patients belonged to three families in which siblings were also affected (P2–P3, P8–P9, and P13–P14). The median age at symptom onset was 3.5 months, and the median age at first clinical evaluation was 4.0 months (Table 1). At the time of the initial examination, 64% of patients presented with hepatomegaly, and 73% had a family history of siblings with similar clinical manifestations. Hypoglycemia was documented in 4 of 14 patients (28.6%), while metabolic acidosis occurred in 4 of 7 evaluated cases (57.1%). All patients with available laboratory data demonstrated elevated liver enzymes and increased blood lactate levels (13/13; 100%). Among patients who underwent lipid profiling, 9 of 11 (81.8%) showed hypertriglyceridemia, and 7 of 11 (63.6%) had elevated total cholesterol concentrations. Evidence of growth retardation was observed in 3 of 5 patients (60%).

**Table 1:** Summary of clinical and biochemical characteristics of 24 patients with glycogen storage disease I.

	Phenotype		Total n (%)	Type Ia n (%)	Type Ib n (%)	*p*
Clinical symtoms	Age of onset (mo) (Median, Min-Max)		4 (1–24)	3.5 (1−12)	5 (1–24)	0.318⁎
	Age of first visit (mo) (Median, Min-Max)		5 (1–138)	4.0 (1–138)	6 (1–82)	0.482⁎
	Gender (*n* = 24)	Male	14 (58.3)	8 (53.3)	6 (66.7)	0.678⁎⁎
		Female	10 (41.7)	7 (46.7)	3 (33.3)	
	Family History (n = 24)	Yes	15 (62.5)	11(73.3)	4 (44.4)	0.212⁎⁎
		No	9 (37.5)	4 (26.7)	5 (55.6)	
	Hepatomegaly (n = 23)	Yes	16 (69.6)	9 (64.3)	7 (77.8)	0.657⁎⁎
		No	7 (30.4)	5 (35.7)	2 (22.2)	
	Doll-like facies (*n* = 18)	Yes	8 (44.4)	5 (41.7)	3 (50)	1.00⁎⁎
		No	10 (55.6)	7 (58.3)	3 (50)	
	Growth delay (height < −2 SDS) (*n* = 6)	Yes	4 (66.7)	3 (60)	1 (100)	1.00⁎⁎
		No	2 (33.3)	2 (40)	0 (0)	
Biochemical features	pH < 7.35 (*n* = 8)	Yes	4 (50)	4 (57.1)	0 (0)	1.00⁎⁎
		No	4 (50)	3 (42.9)	1 (100)	
	Hypoglycemia (*n* = 22)	Yes	5 (22.7)	4 (28.6)	1 (12.5)	0.613⁎⁎
		No	17 (77.3)	10 (71.4)	7 (87.5)	
	Elevated transaminases (n = 24)	Yes	24 (100)	15 (100)	9 (100)	1.00⁎⁎
		No	0 (0)	0 (0)	0 (0)	
	Hypercholesterol (*n* = 17)	Yes	7 (41.2)	7 (63.6)	0 (0)	0.035**
		No	10 (58.8)	4 (36.3)	6 (100)	
	Hypertriglycerid (*n* = 17)	Yes	15 (88.2)	9 (81.8)	6 (100)	0.515⁎⁎
		No	2 (11.8)	2 (18.2)	0 (0)	
	Hyperlactat (n = 22)	Yes	22 (100)	13 (100)	9 (100)	1.00⁎⁎
		No	0 (0)	0 (0)	0 (0)	
	Neutropenia (*n* = 23)	Yes	4 (17.4)	0 (0)	4 (50)	***0.008***⁎⁎
		No	19 (82.6)	15 (100)	4 (50)	

⁎ : Mann–Whitney U test.

⁎⁎ : Fisher's Exact Test.

The GSD Ib subgroup included nine patients from seven unrelated families, among whom two sibling pairs were identified (P16–P17 and P22–P23). The median age at symptom onset was 5 months, whereas the median age at the first clinical evaluation was 6 months (Table 1). Hepatomegaly was present in 77.8% of patients, and 44.4% reported a positive family history. Among the eight patients whose biochemical parameters were assessed at the initial evaluation, all showed elevated liver enzymes (100%), whereas hypoglycemia was observed in one patient (12.5%). In addition, all patients with available laboratory data exhibited elevated blood lactate and triglyceride levels (100%). In contrast, none of the six patients with complete lipid measurements demonstrated hypercholesterolemia, which was significantly different from patients with GSD Ia (63.6% vs. 0%, *p* = 0.035). Notably, neutropenia was identified in half of the GSD Ib patients at the time of initial clinical assessment, which showed a significantly difference compared with GSD Ia patients (0% vs. 50%, *p* = 0.008).

### 3.2 Genotype

**Fig. 1:**
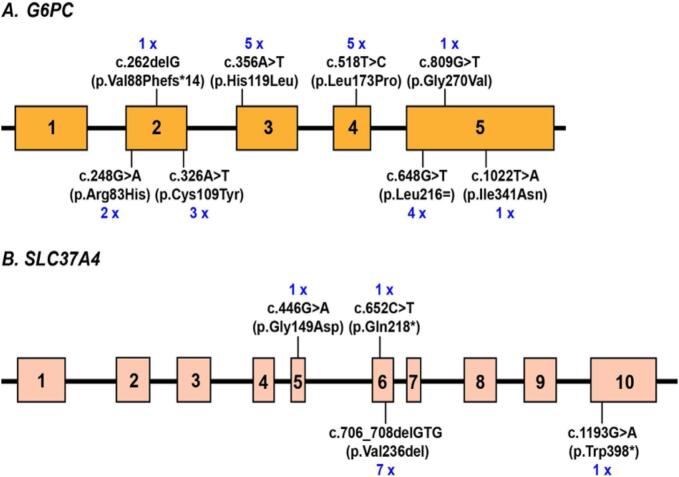
Location of variants in the *G6PC (A)* and *SLC37A4 (B*) genes identified in 24 patients with glycogen storage disease type I. The blue number indicates the number of patients with the corresponding variants.

Among patients with GSD Ia (*n* = 15), pathogenic/likely pathogenic variants in the *G6PC* gene were identified in all individuals. Of these, eight patients carried homozygous variants, whereas seven patients exhibited compound heterozygous variants. A total of eight distinct *G6PC* variants were detected, comprising one synonymous variant, one frameshift variant, and six missense variants (Fig. 1A). All identified variants have been previously reported in the literature (Table 2). The genotypes of patients P6 and P11 were previously described in Vietnamese publications; however, those reports focused only on clinical manifestations and genotypic findings at the time of diagnosis. In the GSD Ib cohort (*n* = 9), all patients carried pathogenic or likely pathogenic variants in the *SLC37A4* gene. Among them, eight patients showed homozygosity, while one patient presented with a compound heterozygous status. Four distinct variants were identified within this group, including two missense variants, one nonsense variant, and one in-frame deletion (Fig. 1B).

**Table 2:** Genotypic characteristics of 24 patients with with glycogen storage disease I.

Patients	Gene	Genotype	c.DNA change	AA change	Effect	dbSNP	Clinvar
GSD Ia							
01 (P1)	*G6PC*	CH	c.518 T > C	p.Leu173Pro	Missense	rs775826449	Pathogenic 640,818
			809G > T	p.Gly270Val	Missense	rs80356483	Pathogenic 21,063
02 (P2, P3)	*G6PC*	CH	c.248G > A	p.Arg83His	Missense	rs1801176	Pathogenic 38,300
			c.356 A > T	p.His119Leu	Missense	rs1401928680	Pathogenic 863,144
03 (P4, P10, P11)	*G6PC*	Hom	c.356 A > T	p.His119Leu	Missense	rs1401928680	Pathogenic 863,144
03 (P5, P6, P7)	*G6PC*	Hom	c.518 T > C	p.Leu173Pro	Missense	rs775826449	Pathogenic 640,818
02 (P8, P9)	*G6PC*	Hom	c.648G > T	p.Leu216=	Synonymous	rs80356484	Pathogenic 12,003
01 (P12)	*G6PC*	CH	c.262delG	p.Val88PhefsTer14	Frameshift	rs755612674	Pathogenic 643,470
			c.518 T > C	p.Leu173Pro	Missense	rs775826449	Pathogenic 640,818
02 (P13, P14)	*G6PC*	CH	c.648G > T	p.Leu216=	Synonymous	rs80356484	Pathogenic 12,003
			c.326G > A	p.Cys109Tyr	Missense	rs886052955	Pathogenic 323,360
01 (P15)	*G6PC*	CH	c.326G > A	p.Cys109Tyr	Missense	rs886052955	Pathogenic 323,360
			c.1022 T > A	p.Ile341Asn	Missense	rs387906505	Pathogenic 12,009

GSD Ib							
06 (P16, P17, P18, P21, P22, P23)	*SLC37A4*	Hom	c.706_708 delGTG	p.Val236del	In-frame deletion	rs121908977	Pathogenic 552,882
01 (P19)	*SLC37A4*	Hom	c.1193G > A	p.Trp398Ter	Nonsense	Novel	Pathogenic according to ACMG classification (PVS1, PM2, PM3, PP3, PP4)
01 (P20)	*SLC37A4*	Hom	c.446G > A	p.Gly149Asp	Missense	rs193302892	Pathogenic 68,280
01 (P24)	*SLC37A4*	CH	c.706_708 delGTG	p.Val236del	In-frame deletion	rs121908977	Pathogenic 552,882
			c.652C > T	p.Gln218Ter	Nonsense	rs551439289	Pathogenic 189,147

AA, amino acid; ACMG, American College of Medical Genetics and Genomics; CH, compound heterozygous; cDNA, complementary DNA; dbSNP, database of single nucleotide polymorphisms; Hom, homozygous; PVS, pathogenic very strong; PM, pathogenic moderate; PP, pathogenic support.

**Fig. 2:**
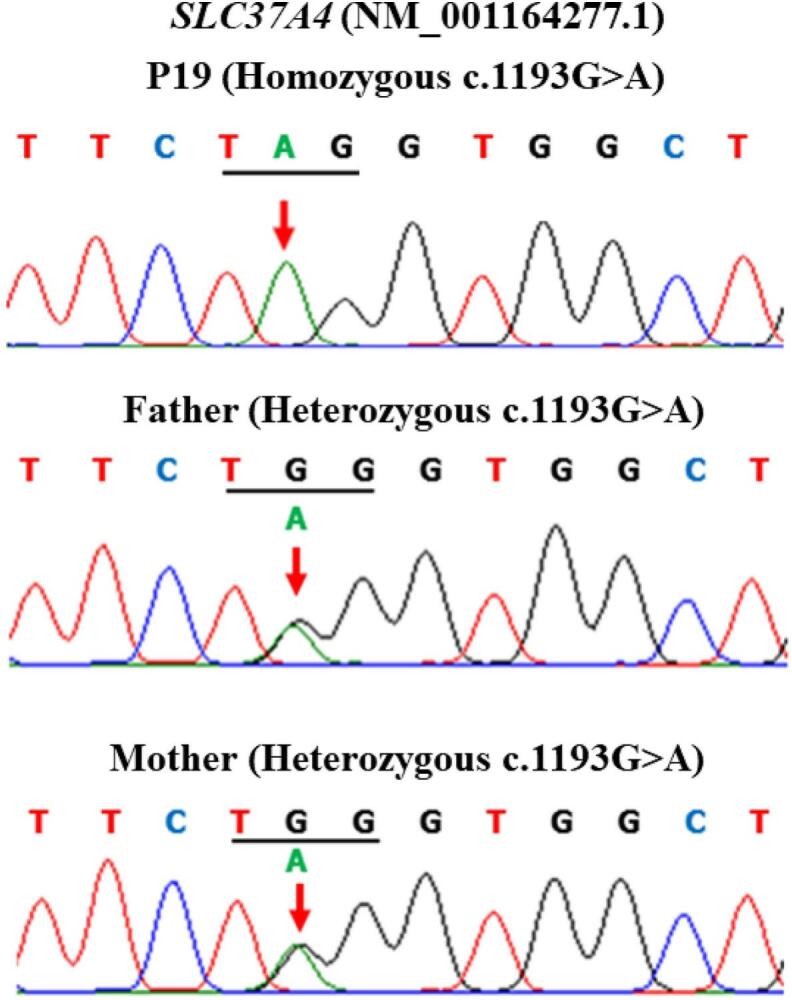
Chromatogram of Sanger sequencing results from family of patient P19. Patient P19 was homozygous for the *SLC37A4*: c.1193G > A variant, which was inherited from both the father and the mother. This variant represents a novel mutation that has not been previously reported.

In addition, our study identified a previously unreported variant involving a nucleotide substitution at position 1193 of *SLC37A4*, in which guanine (G) is replaced by adenine (A). This sequence alteration introduces a premature stop codon at amino acid position 398 of the SLC37A4 protein. This newly identified variant was classified as pathogenic based on the criteria established by the ACMG variant classification guidelines (PVS1, PM2, PM3, PP3, and PP4) (Table 2). The patient carrying the novel variant was a female infant (P19) from a family with two children and no prior history of metabolic disease. She required hospitalization during infancy due to a postpartum infection, and laboratory evaluation revealed elevated liver enzyme levels and increased blood lactate concentrations. Molecular diagnosis was subsequently confirmed by genetic analysis and Sanger sequencing, which demonstrated that both parents were heterozygous carriers of the variant (Fig. 2).

### 3.3 Treatment and outcome

Initial management primarily consisted of dietary therapy, including frequent feeding, restriction of fructose-, sucrose-, and lactose-containing foods, and administration of uncooked cornstarch (UCCS). UCCS therapy was initiated after 12 months of age in most patients. Detailed treatment characteristics are summarized in Table 3. No patients received continuous nocturnal enteral feeding during initial management.

**Table 3:** Initial dietary management and metabolic treatment strategies at diagnosis in Vietnamese patients with GSD I.

Pt	Initial diagnosis	Initial hospitalization	Age at diagnosis (months)	UCCS initiation	Feeding interval	Dietary modification
GSD Ia						
P1	GSD	No	14	Yes (1 g/kg/dose)	q4h	Lactose-free formula (Isomil)
P2	Meningitis	Yes	5	No	q2-3h	Lactose-free formula (Isomil)
P3	GSD	Yes	1	No	q2-3h	Lactose-free formula (Isomil)
P4	Acute respiratory failure	Yes	4	No	q2-3h	Lactose-free formula (Isomil)
P5	GSD	Yes	4	No	q2-3h	Lactose-free formula (Isomil)
P6	Persistent diarrhea	No	4	No	q2-3h	Lactose-free formula (Isomil)
P7	Bronchiolitis	Yes	4	No	q2-3h	Lactose-free formula (Isomil)
P8	Unexplained hepatomegaly	Yes	8	No	q2-3h	Lactose-free formula (Isomil)
P9	Fatty acid oxidation disorders	Yes	10	No	q2-3h	Lactose-free formula (Isomil)
P10	Neonatal infection	Yes	1	No	q2-3h	Lactose-free formula (Isomil)
P11	GSD	No	3	No	q2-3h	Lactose-free formula (Isomil)
P12	GSD	Yes	12	Yes (1 g/kg/dose)	q4h	Lactose-free formula (Isomil)
P13	Hepatomegaly - suspected metabolic disorder	Yes	138	Yes (2 g/kg/dose)	q6h	Lactose-free formula (Isomil)
P14	Persistent diarrhea	No	7	No	q2-3h	Lactose-free formula (Isomil)
P15	Persistent diarrhea	No	8	No	q2-3h	Lactose-free formula (Isomil)

GSD Ib						
P16	GSD	No	29	Yes (2 g/kg/dose)	q4h	Lactose-free formula (Isomil)
P17	GSD	No	2	No	q2-3h	Lactose-free formula (Isomil)
P18	GSD	No	6	No	q2-3h	Lactose-free formula (Isomil)
P19	Neonatal infection	Yes	1	No	q2-3h	Lactose-free formula(Isomil)
P20	Pneumonia	Yes	4	No	q2-3h	Lactose-free formula (Isomil)
P21	GSD	Yes	10	No	q2-3h	Lactose-free formula (Isomil)
P22	GSD	No	82	Yes (2 g/kg/dose)	q6h	Lactose-free formula (Isomil)
P23	GSD	No	70	Yes (2 g/kg/dose)	q4-6h	Lactose-free formula (Isomil)
P24	Poor feeding and failure to thrive	No	10	No	q2-3h	Lactose-free formula (Isomil)

GSD, Glycogen storage diseases; UCCS, uncooked cornstarch; q2–3h, feeding every 2–3 h; q4h, feeding every 4 h; q4–6h, feeding every 4–6 h; q6h, feeding every 6 h; No, not used/not performed; Yes, used/performed.

The duration of follow-up ranged from 1.5 to 13 years. Regular specialized follow-up was documented in 14/15 patients with GSD Ia and 4/9 patients with GSD Ib. Inadequate UCCS administration (≤1–2 times/day) was recorded in 15/24 patients during follow-up.

Long-term complications were observed in both subgroups. Among patients with GSD Ia, hepatic adenoma was identified in 3/15 patients, liver cirrhosis in 2/15, and acute pancreatitis in 1/15. Two patients with GSD Ia died during follow-up. In the GSD Ib subgroup, all patients developed neutropenia, while recurrent infections were documented in 5/9 patients. Additional complications included pulmonary arterial hypertension, Moyamoya-like syndrome, nephrolithiasis, and inflammatory bowel disease-like manifestations. Growth impairment was observed in both groups, with height-for-age SDS ranging from −7.7 to −2.0 in patients with GSD Ia and from −7.4 to −2.3 in patients with GSD Ib. Long-term treatment adherence, complications, and overall outcomes are summarized in Table 4, while detailed patient-level follow-up characteristics and individual outcomes are presented in Supplementary Table 4.

**Table 4:** Treatment adherence, follow-up characteristics, complications, and outcomes in patients with GSD I.

Variable	GSD Ia (n = 15)	GSD Ib (n = 9)	Total (n = 24)
Follow-up duration, years (range)	1.5–13	1.5–12.5	1.5–13
Regular specialized follow-up	14/15 (93.3%)	4/9 (44.4%)	18/24 (75.0%)
Interrupted specialized follow-up	1/15 (6.7%)	4/9 (44.4%)	5/24 (20.8%)
Inadequate UCCS administration (≤1–2×/day)	9/15 (60.0%)	6/9 (66.7%)	15/24 (62.5%)

Major complications			
Hepatic adenoma	3/15 (20.0%)	0	3/24 (12.5%)
Liver cirrhosis	2/15 (13.3%)	0	2/24 (8.3%)
Acute pancreatitis	1/15 (6.7%)	0	1/24 (4.2%)
Recurrent infections	0	5/9 (55.6%)	5/24 (20.8%)
Pulmonary arterial hypertension	1/15(6.7%)	1/9 (11.1%)	2/24 (8.3%)
Moyamoya syndrome	0	1/9 (11.1%)	1/24 (4.2%)
Nephrolithiasis	0	1/9 (11.1%)	1/24 (4.2%)
Death	2/15 (13.3%)	0	2/24 (8.3%)
Height-for-age SDS, range	−7.7 to −2.0	−7.4 to −2.3	−7.7 to −2.0

## 4 Discussion

This study provides the first comprehensive description of the clinical and molecular characteristics of Vietnamese patients with GSD I. The findings reveal both notable similarities with other East Asian cohorts and unique regional features within the Vietnamese population.

### 4.1 Clinical features

The age at symptom onset observed in our cohort differed slightly from that reported in a large European multicenter study that included 288 patients with GSD I, of whom 231 had GSD Ia. That study documented a median onset age of 6 months for GSD Ia, whereas in our cohort, the median onset age was 3.5 months. In contrast, patients with GSD Ib in our study showed a median onset age of 5 months, compared with 4 months reported in the same European dataset [6].

With respect to demographic characteristics, the male-to-female ratio in our cohort was 1.4. Notably, approximately two-thirds of the patients had a family history of siblings who had a similar condition. Anthropometric data at diagnosis were available for 25% of patients, among whom 66.7% exhibited short stature. This proportion is higher than the 25% prevalence reported in the European cohort, although interpretation should be cautious given the limited sample size in our study [6]. At the time of diagnosis, hepatomegaly was present in 70% of patients, slightly lower than the 83% prevalence reported in the European study, which assessed abdominal distension as a proxy for liver enlargement [6]. In addition, doll-like facies, a characteristic feature of GSD I, were observed in 44.4% of our patients.

Regarding metabolic abnormalities, among the 22 patients with complete clinical data at initial admission, five presented with hypoglycemia, while metabolic acidosis was documented in in four of eight patients for whom relevant laboratory data were available. Lipid abnormalities also differed between subtypes. Among patients with GSD Ib, all individuals showed elevated triglyceride levels, whereas hypercholesterolemia was not observed. In contrast, 81.8% of patients with GSD Ia had hypertriglyceridemia, and 63.6% exhibited elevated cholesterol concentrations. Importantly, all patients in both subgroups demonstrated elevated transaminase levels and increased blood lactate concentrations, reflecting the underlying disruption of hepatic glucose metabolism characteristic of the disease. Approximately 50% of patients with GSD Ib typically present with neutropenia at the time of initial clinical evaluation.

### 4.2 Genotypic spectrum and geographic characteristics

The genetic findings in our cohort were fully consistent with the known molecular basis of GSD I. Within the GSD Ia subgroup, three recurrent variants were identified in *G6PC*: c.518 T > C (p.Leu173Pro), c.356 A > T (p.His119Leu), and c.648G > T (p.Leu216=). The c.518 T > C variant was detected in eight alleles across five unrelated families. Several patients carried the variant either in the homozygous state or as part of a compound heterozygous genotype, which explains why the number of affected alleles exceeded the number of individuals carrying the mutation. Among these cases, patient P6, previously described by Thu et al. (2022), was a six-month-old Vietnamese boy homozygous for c.518 T > C, whose parents were confirmed as heterozygous carriers through three-generation segregation analysis [25]. The detection of this variant in several independent families underscores its pathogenic relevance and suggests that it represents a recurrent variant in the Vietnamese population, extending earlier sporadic observations reported in Chinese cohorts [26]. The c.356 A > T variant was identified in five patients from four unrelated families, including one pair of affected siblings and one homozygous case previously reported by Nguyen et al. (2020) [27]. Another frequently observed variant, c.648G > T, is a synonymous substitution known to disrupt mRNA splicing. This variant has been recognized as a founder mutation in East Asian populations, with particularly high frequencies reported in Chinese, Japanese, and Korean cohorts [11], [12]. Taken together, these observations suggest that c.518 T > C and c.356 A > T may represent recurrent population-specific variants in Vietnam, whereas c.648G > T reflects a broader East Asian mutational hotspot. Identification of such recurrent variants is clinically important because it can facilitate population-adapted genetic screening strategies and contribute to expanding the global mutational spectrum of GSD Ia.

Previous studies have also demonstrated that the mutation spectrum of *SLC37A4* in GSD Ib varies substantially across geographic regions. In our cohort, the c.706_708delGTG (p.Val236del) variant was the most frequently detected mutation, accounting for 72% of alleles, which suggests a potential founder effect in the Vietnamese population. By comparison, other variants predominate in different populations worldwide. For example, c.1042_1043delCT (p.Leu348Valfs) has been reported as the most common variant in mixed Caucasian and German cohorts [28], [29], [30], c.443C > T (p.Ala148Val) is predominant in Korean patients [31], c.352 T > C (p.Trp118Arg) is the dominant variant among Japanese patients [16], and c.572C > T (p.Pro191Leu) is the most frequently reported mutation in Chinese cohorts [29]. Such results emphasize population-specific differences in mutation frequencies. Consequently, the systematic collection of regional genetic data is essential to improve the accuracy of molecular diagnosis, carrier detection, and genetic counseling, particularly in underrepresented Southeast Asian populations.

Comparing the variants discovered in our cohort with global data, we obtained the results shown in Table 5.

**Table 5:** Comparison of *G6PC* and *SLC37A4* variants identified in Vietnamese patients with those reported worldwide.

Gene	Variant (HGVS c.)	Protein change	Exon	Vietnam (n)	Reported in other populations	Novelty / status	Protein level	References
*G6PC* (GSD Ia)	c.518 T > C	p.(Val173Ala)	4	8/30	Sporadic reports in Chinese cohorts, Vietnames	Case reports	–	[25], [26], [30]
	c.356 A > T	p.(His119Leu)	3	8/30	Vietnames, Chinese	Case report	45.78	[27], [32], [33]
	c.648G > T	p.(Leu216Leu)	5	6/30	China (54–83%); Japan (86–91%) Korea (75%) Vietnames (case report)	Common East Asian hotspot	52.25	[3], [9], [11], [12], [32], [34], [35]
	c.326G > A	p.(Cys109Tyr)	2	3/30	Chinese	Case report	14.85	[32], [36]
	c.248G > A	p.(Arg83His)	2	2/30	Common in Chinese	Recurrent	28.46	[10], [11], [32]
	c.262delG	Frameshift (p.Val88PhefsTer14)	2	1/30	Chinese	Case reports	–	[34], [37]
	c.809G > T	p.(Gly270Val)	5	1/30	Iran, French	Case report	–	[3], [38], [39]
	c.1022 T > A	p.(Leu341Gln)	5	1/30	Chinese, Taiwan	Case report	–	[3], [40], [41]
*SLC37A4* (GSD Ib)	c.706_708delGTG	In-frame deletion (Val236del)	6	13/18	Liban	Case report	–	[42]
	c.446G > A	p.(Gly149Glu)	3	2/18	China, Brazil	–	–	[30], [43], [44]
	c.652C > T	p.(Gln218Ter)	5	1/18	Caucasian	–	–	[10]
	c.1193G > A	p.(Trp398Ter)	10	2/18	–	Novel	–	–

### 4.3 Treatment challenges and long-term outcomes

Although international guidelines for GSD I emphasize strict dietary management with frequent feeding, regular administration of uncooked cornstarch (UCCS), and avoidance of prolonged fasting, achieving these treatment goals remained challenging throughout much of the study period in our cohort. Contemporary recommendations generally advocate individualized daytime UCCS administration every 3–5 h after infancy, together with restriction of fructose-, sucrose-, and galactose-containing foods, while total carbohydrate intake should be adjusted according to age, body weight, metabolic control, and growth status. In infants whose metabolic control cannot be maintained with oral feeding alone, continuous nocturnal enteral feeding is recommended to prevent fasting hypoglycemia.

At our center, dietary recommendations were developed in accordance with internationally accepted principles whenever feasible. Patients and caregivers were advised to consume frequent meals, avoid prolonged fasting, restrict fructose-, sucrose-, and galactose-containing foods, and initiate UCCS after approximately 12 months of age. UCCS was prescribed at an initial dose of approximately 1–2 g/kg per dose, and the dose was subsequently adjusted according to metabolic control and fasting tolerance. However, implementation of these recommendations was often limited by several real-world factors, including delayed referral to specialized metabolic care, limited availability of experienced metabolic dietitians during the earlier years of the study period, financial burden, caregiver adherence, and the absence of advanced nutritional support such as Glycosade and routine nocturnal enteral feeding. In Vietnam, several unique socio-cultural and practical factors may have heavily affected long-term adherence to dietary therapy. The traditional Vietnamese diet is primarily based on rice as the main carbohydrate source, and meals are often prepared using conventional cooking methods, such as boiling, steaming, stir-frying, or deep-frying, and shared within families. These characteristics may make it difficult to integrate the regular use of uncooked cornstarch into daily life. In addition, integrating a strict UCCS schedule every 3–5 h into regular school routines and peer environments remains a major challenge for pediatric patients, often leading to omitted daytime doses, as children generally prefer to align their eating habits with those of their peers. Furthermore, in certain areas, obtaining high-quality UCCS remains difficult, while the practical demands of overnight UCCS administration and the necessary routine adjustments for overnight dosing continue to pose additional challenges for many families, further compromising long-term adherence to dietary therapy. Consequently, although UCCS was prescribed according to standard recommendations whenever possible, long-term adherence was frequently suboptimal. More than 60% of patients received UCCS only once or twice daily during follow-up, a frequency substantially lower than current international standards and unlikely to provide adequate metabolic control.

These treatment limitations almost certainly contributed to the relatively high burden of long-term complications observed in our cohort. Current evidence demonstrates that patients receiving optimized metabolic management, including appropriate UCCS dosing, frequent dietary monitoring, multidisciplinary metabolic care, and regular surveillance, and, more recently, individualized therapy supported by continuous glucose monitoring and extended-release cornstarch where available, generally experience substantially lower rates of hepatic adenoma, pancreatitis, renal complications, and other long-term sequelae than were reported historically [45], [46], [47], [48]. These contemporary management strategies also aim to reduce treatment burden, improve adherence, increase fasting tolerance, and enhance health-related quality of life while maintaining stable metabolic control [49]. Therefore, the complication profile observed in the present study should not be interpreted as representative of the natural history of GSD I under contemporary management. Instead, our findings likely reflect the consequences of prolonged suboptimal metabolic control during a period when specialized metabolic services were still developing in Vietnam.

The association between inadequate metabolic control and adverse outcomes was particularly evident in our cohort. Growth impairment remained one of the most common long-term findings, and several patients with the most severe short stature had persistently inadequate UCCS administration throughout follow-up. Similarly, hepatic adenomas, liver cirrhosis, and acute pancreatitis occurred predominantly in patients with prolonged metabolic instability. Persistent hypertriglyceridemia is a well-recognized risk factor for hepatic adenoma complications, emphasizing the importance of sustained metabolic control rather than disease subtype alone [50]. Likewise, the two deaths observed in our cohort occurred among patients treated during earlier periods of the study, when access to specialized metabolic care was considerably more limited than at present.

Recent studies suggest that the sodium–glucose cotransporter-2 (SGLT2) inhibitor empagliflozin may represent a promising targeted therapy for patients with Glycogen Storage Disease Type Ib, particularly for the management of neutropenia and neutrophil dysfunction. International expert recommendations propose initiating empagliflozin therapy in patients with GSD Ib who exhibit clinical manifestations or laboratory evidence of neutropenia or impaired neutrophil function. The recommended starting dosage is approximately 0.1–0.9 mg/kg/day, administered once daily in the morning. These recommendations are supported by accumulating clinical evidence demonstrating improvements in neutrophil counts and function following treatment with SGLT2 inhibitors [51], [52]. No patients in this cohort received granulocyte colony-stimulating factor (G-CSF) during follow-up. In our cohort, all patients with GSD type Ib demonstrated neutropenia on laboratory testing. Among these individuals, four patients had already developed inflammatory bowel disease (IBD), whereas one patient had a history of recurrent oral ulcers. Based on current therapeutic recommendations, empagliflozin was initiated at 0.3 mg/kg once daily after breakfast, and no patient received a dose exceeding 10 mg. At the initiation of therapy, patients were admitted for inpatient observation to monitor the risk of hypoglycemia and potential adverse effects. Following this initial monitoring period, treatment was continued on an outpatient basis with regular follow-up visits. Data regarding treatment-related adverse events are currently being monitored and continue to be collected for further evaluation.

Despite these treatment-related limitations, the present study remains clinically important because it provides the first comprehensive genotype–phenotype characterization of Vietnamese patients with GSD I. The identification of recurrent regional variants may facilitate molecular diagnosis and genetic counseling, whereas the long-term follow-up data highlight important healthcare gaps that remain relevant for many resource-limited settings. Rather than reflecting the expected prognosis of patients receiving contemporary management, our findings underscore the need to strengthen multidisciplinary metabolic services, improve long-term dietary education, increase adherence to recommended UCCS regimens, expand access to continuous glucose monitoring and specialized nutritional support, and facilitate earlier referral to metabolic centers. Implementation of these strategies is likely to improve long-term metabolic control and substantially reduce preventable complications among children with GSD I in Vietnam.

### 4.4 Study limitations

This study has several limitations. First, the retrospective single-center design and relatively small sample size may limit the generalizability of the findings. Second, follow-up duration, treatment adherence, and biochemical monitoring varied among patients because the cohort included individuals managed over an extended period during which specialized metabolic care systems in Vietnam were still evolving.

In addition, several components of contemporary metabolic care, including continuous glucose monitoring (CGM), Glycosade, and dedicated multidisciplinary metabolic support, were not consistently available during much of the study period. Delayed referral to specialized metabolic centers and interruptions in long-term follow-up, particularly during the COVID-19 pandemic, may also have influenced treatment adherence, long-term metabolic control, and complication outcomes in some patients. These delays may reflect geographic barriers, socioeconomic burden, and historical limitations in access to molecular diagnosis and specialized metabolic care in Vietnam.

Despite these limitations, this study represents the first comprehensive characterization of Vietnamese patients with GSD I and provides important real-world data regarding clinical presentation, molecular spectrum, treatment challenges, and long-term outcomes in a resource-limited setting.

## 5 Conclusion

Vietnamese patients with Glycogen Storage Disease Type I exhibit phenotypic and genotypic features that are largely consistent with those reported in other East Asian populations. The variants *G6PC*: c.518 T > C, c.356 A > T, and c.648G > T appear to represent recurrent or relatively frequent mutations in Vietnam, whereas *SLC37A4*: c.706_708delGTG predominates among cases of GSD Ib. The long-term management and follow-up of these patients remain challenging, as reflected by poor treatment adherence and a substantial burden of complications. These findings also suggest the presence of a possible regional founder effect. There is therefore an urgent need to implement targeted strategies for early diagnosis, comprehensive clinical management, and structured long-term follow-up in order to improve outcomes for affected children in Vietnam.

## CRediT authorship contribution statement

**Hang Thi Nguyen:** Conceptualization, writing-review & editing, writing-original draft, software, investigation, formal analysis, data curation; **Dung Chi Vu:** Supervision, methodology, formal analysis, software; **Tung Viet Cao:** Investigation, resources; **Van Khanh Tran:** Supervision, investigation, data curation, validation; **Thao Phuong Bui:** Formal analysis, visualization; **Ngoc Bich Thi Can**: Resources, visualization; **Lan Hoang Nguyen:** Investigation, formal analysis; **Ngoc Lan Nguyen:** Validation, writing – review & editing; **Thinh Huy Tran:** Investigation, project administration; **Nguyen Huu Tu:** Investigation, conceptualization**; Le Thi Phuong:** Investigation, software; **Khanh Ngoc Nguyen:** Writing-review & editing, methodology, data curation, conceptualization, funding acquisition.

## Patient consent statement

Written informed consent was obtained from the parents of the individuals to participate the study and publish this paper.

## Ethics approval

The study protocol was approved by the VNCH Ethics Committee. All procedures were performed in accordance with the principles outlined in the Declaration of Helsinki (IRB: VN01037/IRB00011976/FWA00028418).

## Funding

This research was funded by the Vietnam Ministry of Science and Technology under grant number ĐTĐL.CN.133/21.

## Declaration of competing interest

The authors declare that they have no conflicts of interest.

## Data Availability

The data supporting this study's findings are available from the corresponding author upon reasonable request.
