# Supplementary material for: Genotype–phenotype spectrum and clinical outcomes of glycogen storage disease type I: A 15-year experience at Vietnam National Children's Hospital
Source: Mol Genet Metab Rep. 2026 Jul 10;48:101337. doi: 10.1016/j.ymgmr.2026.101337 (PMC13380477; doi:10.1016/j.ymgmr.2026.101337)
Supplement: Supplementary file 1 — Supplementary material [file mmc1.docx]

**Supplementary data**

**Supplementary Table 1: Individual patient characteristics at the first clinical evaluation.**

| **Case** | **Age at onset (mo)** | **Time of first visit (mo)** | **Sex** | **Hepatomegaly** | **Doll-like facies** | **Height SDS** | **Glucose** | **GOT** | **GPT** | **Uric** | **Lactat** | **pH** | **Cholesterol** | **Triglycerid** | **Neutropenia** | **Family history** |
| --- | --- | --- | --- | --- | --- | --- | --- | --- | --- | --- | --- | --- | --- | --- | --- | --- |
| **GSD Ia** | | | | | | | | | | | | | | | | |
| P1 | 3.5 | 14 | M | + | + | NA | 1.2 | 3240.2 | 1426 | NA | 24.7 | 7.03 | NA | NA | - | + |
| P2 | 4 | 4 | F | + | NA | NA | 2.59 | 2660.7 | 779.5 | NA | 22.01 | NA | 5.43 | 5.92 | - | + |
| P3 | 1 | 1 | M |  | - | -0.1 | 3.9 | 111.3 | 26 | NA | 11.63 | 7.37 | 1.31 | 0.4 | - | + |
| P4 | 3 | 3 | F | - | - | NA | 6.55 | 176.7 | 51.4 | NA | 8.12 | NA | 3.78 | 2.34 | - | - |
| P5 | 4 | 4 | M | + | - | -1.5 | 2.44 | 429.8 | 200.7 | NA | 14.13 | 7.3 | 6.16 | 6.72 | - | + |
| P6 | 2 | 4 | M | - | - | NA | 6.53 | 179.6 | 105.7 | NA | 4.39 | 7.37 | NA | NA | - | - |
| P7 | 3 | 4 | M | + | - | NA | 4.6 | 220.4 | 141.9 | 551.7 | 8.26 | 7.34 | 5.7 | 15.47 | - | + |
| P8 | 6 | 8 | F | + | NA | NA | 5.34 | 278.8 | 102.5 | NA | NA | NA | 6.6 | 6.52 | - | + |
| P9 | 1 | 10 | F | + | + | NA | 4.26 | 253.73 | 2.01 | NA | NA | NA | 5.11 | 10.56 | - | + |
| P10 | 1 | 1 | F | - | - | NA | NA | 256.1 | NA | NA | 5.9 | NA | NA | NA | - | + |
| P11 | 3 | 3 | M | NA | NA | NA | 1.9 | NA | 400 | NA | 9.5 | 7.05 | NA | NA | - | + |
| P12 | 12 | 12 | F | + | + | -2.3 | 3.7 | 584.70 | 445.60 | 392.00 | 13.70 | 7.42 | 2.07 | 8.33 | - | - |
| P13 | 8 | 138 | M | + | + | -5.6 | 7.5 | 162.50 | 125.40 | 1176.20 | 21.02 | NA | 6.49 | 18.4 | - | + |
| P14 | 7 | 7 | F | + | + | -2.7 | 4.58 | 126.50 | 140.70 | 371.36 | 8.58 | NA | 5.52 | 12 | - | + |
| P15 | 5 | 5 | M | - | - | NA | 4.73 | 331.8 | 98.9 | NA | 6.8 | NA | 5.38 | 17.23 | - | - |
| Median  Min – Max | 3.5  (1-12) | 4.0  (1-138) |  |  |  |  | 4.4  (1.2-7.5) | 254.9 (111-3240) | 133.1  (2-1426) | 551,7  (392-1176) | 9.5  (4.39-24.7) |  | 5.43  (1.31-6.6) | 8.33  (0.4-18.4) |  |  |
| **GSD Ib** | | | | | | | | | | | | | | | | |
| P16 | 4 | 29 | M | + | + | NA | NA | 60 | NA | NA | 8.2 | NA | NA | NA | - | + |
| P17 | 2 | 2 | M | - | - | NA | 5.0 | 91.1 | 53.1 | 392.3 | 7.14 | NA | 3.24 | 3.02 | + | + |
| P18 | 6 | 6 | F | + | NA | NA | 5.06 | 165.80 | 31.80 | 335.50 | 4.1 | NA | 2.01 | 3.46 | + | - |
| P19 | 1 | 1 | F | - | - | NA | 2.82 | 97.8 | 80.2 | NA | 13.42 | NA | 3.36 | 6.48 | - | - |
| P20 | 3 | 4 | M | + | NA | NA | 7.8 | 356.50 | 99.70 | NA | 9.98 | NA | 4.0 | 7.82 | - | - |
| P21 | 10 | 10 | F | + | NA | NA | 4.44 | 57.30 | 20.8 | 486.63 | 6.33 | NA | 4.7 | 8.61 | + | - |
| P22 | 12 | 82 | M | + | + | NA | 2.42 | 104.00 | 140.80 | NA | 18.4 | NA | NA | NA | NA | + |
| P23 | 24 | 70 | M | + | + | NA | 5.28 | 510.2 | 195.1 | NA | 7.55 | NA | 3.7 | 5.12 | - | + |
| P24 | 5 | 5 | M | + | - | -2.6 | 3.48 | 189.20 | 153.10 | NA | 5.85 | 7.48 | NA | NA | + | - |
| Median  Min - Max | 5  (1-24) | 6  (1-82) |  |  |  |  | 4.7  (2.4-7.8) | 104  (57.3-510.2) | 90  (20.8-195.1) | 392.3  (335.5-486.6) | 7.6  (4.1-18.4) |  | 3.53  (2.01-4.7) | 5.8  (3.02-8.61) |  |  |
| p* | 0.318 | 0.482 |  |  |  |  | 0.714 | *0.033* | 0.267 | 0.4 | 0.186 |  | *0.048* | 0.216 |  |  |

*: Mann–Whitney U test

SDS, Standard Deviation Score; NA, not avaible

**Supplementary Table 4: Individual treatment characteristics, follow-up adherence, complications, and outcomes of patients with GSD I**

| **Patients** |  | | | | |  | | | | | | **Complications** | | | | | | | | | | | |
| --- | --- | --- | --- | --- | --- | --- | --- | --- | --- | --- | --- | --- | --- | --- | --- | --- | --- | --- | --- | --- | --- | --- | --- |
|  |  |  |  |  |  | **UCCS therapy (times/day)** | **Allopurinol** | **Empagliflozin therapy** | | | | **Seizure** |  | | **Acute pancreatitis** |  | | | | **Height-for-age SDS** | **Nephrolithiasis** | **Recurrent infections** | **Outcome** |
|  | **Age at last evaluation** | **Follow-up duration** | **Follow-up adherence** | **Outpatient visits** | **Hospitalizations** |  |  | **Dose**  **mg/kg/day** | **Age at treatment initiation** | **ANC before treatment** | **ANC**  **at the last evaluation** |  | **Pulmonary arterial hypertension** | **Other cardiovascular manifestations** |  | **Adenoma** | **Liver**  **cirrhosis** | **AFP**  **(ng/mL)** | **Surgical resection** |  |  |  |  |
| P1 | 7y8mo | 6.5y | Lost to follow-up during the last 2 years | 15 | 5 | Not used | - | - | - | - | - | - | - | - | - | - | Cirrhosis | NA | - | NA | NA | - | Died at 9y9mo |
| P2 | 8y3mo | 8y | Regular follow-up | 26 | 10 | \|  \| \| --- \|   1/day | + | - | - | - | - | - | - | - | - | - | - | - |  | -6.5 | - | - | Alive |
| P3 | 1y5mo | 1.5y | Regular follow-up | 5 | 10 | Not used | - | - | - | - | - | - | - | - | - | - | - | - |  | -4.9 | - | - | Alive |
| P4 | 7y5mo | 7y | Regular follow-up | 30 | 18 | 3/day | + | - | - | - | - | - | - | - | - | Adenoma | - | 1.0 | + | -5.4 | - | - | Alive |
| P5 | 5y | 4.5y | Regular follow-up | 12 | 10 | 3/day | + | - | - | - | - | - | - | - | - | - | - | - | - | -4.3 | - | - | Alive |
| P6 | 6y11mo | 6.5y | Regular follow-up | 24 | 6 | 3-4/day | + | - | - | - | - | - | - | - | - | - | - | - | - | -4.1 | - | - | Alive |
| P7 | 7y5mo | 7y | Regular follow-up | 20 | 23 | 1/day | + | - | - | - | - | - | - | - | - | - | - | - | - | -6.6 | - | - | Alive |
| P8 | 7y1mo | 6y | Regular follow-up | 28 | 2 | 1/day | + | - | - | - | - | - | - | - | - | - | - | - | - | -3.6 | - | - | Alive |
| P9 | 10y7mo | 10y | Regular follow-up | 35 | 2 | 1/day | + | - | - | - | - | - | - | - | - | - | - | - | - | -4.6 | - | - | Alive |
| P10 | 13y11mo | 13y | Regular follow-up | 50 | 3 | 1/day | + | - | - | - | - | - | + | - | - | Adenoma | - | 1.4 | - | -2 | - | - | Alive |
| P11 | 13y7mo | 13y | Regular follow-up | 31 | 13 | 1/day | + | - | - | - | - | - | - | Hypertension | - | - | Cirrhosis | - | - | -7.7 | - | - | Alive |
| P12 | 2y5mo | 1.5y | Regular follow-up | 8 | 5 | 3/day | - | - | - | - | - | - | - | - | - | - | - | - | - | -2.6 | - | - | Alive |
| P13 | 14y2mo | 2.5y | Regular follow-up | 6 | 4 | 1/day | + | - | - | - | - | - | - | - | Acute pancreatitis | Adenoma | - | 1.2 | - | -7.0 | - | - | Alive |
| P14 | 1y9mo | 1y | Regular follow-up | 6 | 2 | 3/day | - | - | - | - | - | - | - | - | - |  | - | - | - | -3.5 | - | - | Alive |
| P15 | 6y6mo | 6y | Followed at local healthcare facilities during the COVID-19 pandemic | 13 | 6 | 3/day | + | - | - | - | - | - | - | - | - | - | - | - | - | -2.9 | - | - | Died from septic shock |
| **GSD Ib** | | | | | | | | | | | | | | | | | | | | | | | |
| P16 | 15y1mo | 12.5y | Non-adherence to dietary regimen during the first 6 years | 44 | 3 | 2-3/day | - | 0.3 | 14y | 0.6 | 2.38 | + | - | - | - | - | - | - | - | -5.6 | - | + (IBD) | Alive |
| P17 | 6y6mo | 6.5y | Regular follow-up | 36 | 5 | 3/day | - | 0.3 | 5y5mo | 0.64 | 1.93 | - | - | - | - | - | - | - | - | -3.0 | - | + (IBD) | Alive |
| P18 | 8y2mo | 7.5y | Regular follow-up | 75 | 32 | 1-2/day | + | - | - | - | - | Moyamoya syndrome | + | - | - | - |  | - | - | -4.4 | - | - | Alive |
| P19 | 10y6mo | 10.5y | Follow-up at local hospitals | 5 | 3 | Not used | - | 0.3 | 10y6mo | 0.34 | 1.83 | - | - | - | - | - | - | - | - | -4.3 | - | +  (Oral ulceration) | Alive |
| P20 | 5y11mo | 5.5y | Followed at a private clinic for 1 year | 13 | 11 | 1-2/day | - | - | - | - | - | - | - | - | - | - | - | - | - | -4.7 | - | - | Alive |
| P21 | 10y3mo | 9.5y | Poor dietary and follow-up adherence during the first 5 years | 56 | 12 | 1-2/day | - | - | - | - | - | - | - | - | - | - | - | - | - | -4.1 | - | - | Alive |
| P22 | 15y11mo | 7y | Follow-up at local hospitals | 12 | 5 | 1/day | + | 0.3 | 15y4mo | 0.77 | 1.47 | - | - | - | - | - | - | - | - | -7.4 | + | + (IBD) | Alive |
| P23 | 10y10mo | 4y | Follow-up at local hospitals | 8 | 2 | 1/day | + | 0.3 | 10y3mo | 0.4 | 1.69 | - | - | - | - | - | - | - | - | -4.3 | - | + (IBD) | Alive |
| P24 | 2y | 1.5y | Regular follow-up | 10 | 3 | 3-4/day | - | - | - | - | - | - | - | - | - | - | - | - | - | -2.3 | - | - | Alive |

Y: year; mo: months; NA: not available; IBD: inflammatory bowel disease; −: none.
